# Galectin-7 as a potential predictive marker of chemo-and/or radio-therapy resistance in oral squamous cell carcinoma

**DOI:** 10.1002/cam4.195

**Published:** 2014-02-07

**Authors:** Sho Matsukawa, Kei-ichi Morita, Ayako Negishi, Hiroyuki Harada, Yusuke Nakajima, Hiroaki Shimamoto, Hirofumi Tomioka, Kae Tanaka, Masaya Ono, Tesshi Yamada, Ken Omura

**Affiliations:** 1 Oral and Maxillofacial Surgery, Department of Oral Health Sciences, Graduate School of Medical and Dental Sciences, Tokyo Medical and Dental University1-5-45 Yushima, Bunkyo-ku, Tokyo, 113-8510, Japan; 2 Department of Advanced Molecular Diagnosis and Maxillofacial Surgery, Hard Tissue Genome Research Center, Tokyo Medical and Dental University1-5-45 Yushima, Bunkyo-ku, Tokyo, 113-8510, Japan; 3 Division of Chemotherapy and Clinical Research, National Cancer Center Research Institute5-1-1 Tsukiji, Chuo-ku, Tokyo, 104-0045, Japan

**Keywords:** Formalin-fixed paraffin-embedded, galectin-7, liquid chromatography and mass spectrometry, oral squamous cell carcinoma

## Abstract

Treatment of advanced oral squamous cell carcinoma (OSCC) requires the integration of multimodal approaches. The aim of this study was to identify predictors of tumor sensitivity to preoperative radiotherapy/chemotherapy for OSCC in order to allow oncologists to determine optimum therapeutic strategies without the associated adverse effects. Here, the protein expression profiles of formalin-fixed paraffin-embedded (FFPE) tissue samples from 18 OSCC patients, termed learning cases, who received preoperative chemotherapy and/or radiotherapy followed by surgery were analyzed by quantitative proteomics and validated by immunohistochemistry in 68 test cases as well as in the 18 learning cases. We identified galectin-7 as a potential predictive marker of chemotherapy and/or radiotherapy resistance, and the sensitivity and specificity of the galectin-7 prediction score (G7PS) in predicting this resistance was of 96.0% and 39.5%, respectively, in the 68 test cases. The cumulative 5-year disease-specific survival rate was 75.2% in patients with resistant prediction using G7PS and 100% in patients with sensitive prediction. In vitro overexpression of galectin-7 significantly decreased cell viability in OSCC cell line. Therefore, our findings suggest that galectin-7 is a potential predictive marker of chemotherapy and/or radiotherapy resistance in patients with OSCC.

Identification of proteins differentially expressed in OSSC samples from patients sensitive or resistant. The samples were processed by LC-MS and analyzed with 2DICAL.

## Introduction

Oral cancer is the sixth most common cancer worldwide, with an annual incidence of ∼275,000 cases. However, unlike many other cancers, the incidence of oral cancer is increasing 1. In Japan, the number of patients diagnosed with oral cancer was 2100 in 1975 and 6900 in 2005, and this number is estimated to increase to 7800 by 2015, when it will represent 1% of all cancer cases and approximately 40% of all head and neck cancer cases 2. Histopathologically, squamous cell carcinoma (SCC) is the most common cancer of the oral cavity, accounting for >90% of all oral cancer cases. Despite recent improvements in multimodal therapies, the survival rate of these patients remains poor because of frequent locoregional and/or distant recurrences. These statistics highlight the urgent need for treatment alternatives 3.

Treatment of advanced head and neck SCC requires the integration of multimodal approaches. Interest in neoadjuvant chemotherapy has recently regenerated because of its survival benefits, particularly when a taxane–cisplatin–fluorouracil regimen is applied instead of the standard cisplatin–fluorouracil regimen 4–6. Usually, tumor response to neoadjuvant chemotherapy predicts its response to radiotherapy. The prognostic indicators of favorable outcome would allow oncologists to make a more rational selection of therapeutic strategies without the unnecessary toxicities of neoadjuvant chemotherapy.

Over the past decade, gene expression in oral SCC (OSCC) has been studied extensively using microarray techniques. However, gene expression is not always correlated with the level of expression of the corresponding protein 7. Furthermore, although the use of fresh material is required for most analytical approaches, human tissue samples are not always available in sufficient quantity. As an alternative, formalin-fixed paraffin-embedded (FFPE) tissue blocks can be routinely collected and stored as samples for research purposes after pathological diagnosis. A method of extracting proteins from FFPE tissues in the form of tryptic peptides was recently developed, and the methodology is compatible with a variety of mass spectrometry (MS)-based proteomics 8.

This study aimed to identify potential predictive markers of chemotherapy and/or radiotherapy resistance in patients with OSCC using quantitative proteomic analysis of FFPE biopsy tissues.

## Patients and Methods

### Patients

This retrospective study included 86 patients diagnosed with resectable OSCC and treated at the Tokyo Medical and Dental University Hospital Faculty of Dentistry (TMDU, Tokyo, Japan) between January 2001 and December 2011 (Table 1). The diagnosis was confirmed by histological examination of tissue biopsies surgically removed from the center of the cancerous tissue. The FFPE samples were fixed in formalin, embedded in paraffin, and stored at room temperature. Thereafter, all patients received preoperative chemotherapy and/or radiotherapy, followed by surgical primary tumor resection with or without neck dissection. After the chemotherapy and/or radiotherapy, treatment outcome was evaluated using the response evaluation criteria in the solid tumors (RECIST) guidelines. Cases of progressive disease (PD) or stable disease (SD) were assigned to the resistant group (Group R), whereas those who achieved a partial response (PR) or complete response (CR) were assigned to the sensitive group (Group S). This protocol was reviewed and approved by the Ethics Committee Board of the TMDU.

**Table 1:** Clinical characteristics and expression profile of galectin-7.

Patient no.	Case	Group	Gender	Age	Primary site	Differentiation	TN	Stage	G7S	G7NL	Prediction score	Pre C/R	Response to therapy
1	Learning	Group R	F	61	Mandibula	Grade 2	T4N1	4A	29	2	−2.78	PF + S/R	NC
2	Learning	Group R	M	60	Maxillary sinus	Grade 3	T3N2c	4C	0	0	−1.97	P/R	NC
3	Learning	Group R	M	50	Floor of mouth	Grade 2	T3N2b	4A	45.5	2	−1.82	S/R	NC
4	Learning	Group R	M	57	Tongue	Grade 1	T2N1	3	48.4	2	−1.65	S/R	NC
5	Learning	Group R	M	79	Tongue	Grade 2	T3N1	3	48.4	2	−1.65	S/R	NC
6	Learning	Group R	M	32	Tongue	Grade 1	T2N2b	4A	31.5	1	−1.38	S/R	NC
7	Learning	Group R	M	57	Tongue	Grade 1	T3N2b	4A	57.7	2	−1.10	PF + S/R	PD
8	Learning	Group R	M	68	Mandibula	Grade 2	T4N2b	4A	44.9	1	−0.61	PF	NC
9	Learning	Group R	M	47	Tongue	Grade 2	T2N0	2	46.6	1	−0.51	PF	NC
10	Learning	Group S	M	72	Floor of mouth	Grade 2	T3N2c	4A	72.5	2	−0.25	S/R	PR
11	Learning	Group S	M	50	Tongue	Grade 1	T2N0	2	34.6	0	0.04	S	PR
12	Learning	Group S	M	82	Tongue	Grade 1	T2N0	2	56.8	1	0.09	S	PR
13	Learning	Group S	M	59	Tongue	Grade 2	T3N0	3	61.6	0	1.61	S/R	PR
14	Learning	Group S	M	61	Tongue	Grade 1	T2N0	2	66.5	0	1.90	S	PR
15	Learning	Group S	M	68	Lower gingiva	Grade 1	T2N0	2	66.6	0	1.90	S/R	PR
16	Learning	Group S	M	76	Lower gingiva	Grade 1	T2N2b	4A	78.7	0	2.60	S	PR
17	Learning	Group S	M	29	Tongue	Grade 2	T2N0	2	80.4	0	2.70	S/R	PR
18	Learning	Group S	M	53	Lower gingiva	Grade 1	T2N1	3	83.3	0	2.87	S	PR
19	Test	Group R	M	58	Mandibula	Grade 2	T4N2b	4A	24.1	2	−3.06	S	NC
20	Test	Group R	M	70	Floor of mouth	Grade 2	T2N2c	4A	4.53	1	−2.95	R	NC
21	Test	Group R	M	57	Maxillary sinus	Grade 2	T4N1	4B	11.4	1	−2.55	P/R	NC
22	Test	Group R	F	66	Maxillary sinus	Grade 2	T3N2b	4A	12.7	1	−2.47	S/R	NC
23	Test	Group R	M	45	Tongue	Grade 3	T3N2b	4A	34.4	2	−2.46	PF	NC
24	Test	Group R	M	60	Tongue	Grade 2	T4N2c	4A	35.3	2	−2.41	S	NC
25	Test	Group R	F	81	Lower gingiva	Grade 3	T4N0	4A	16.6	1	−2.25	R	NC
26	Test	Group R	F	72	Buccal mucosa	Grade 1	T4N2b	4A	0	0	−1.97	R	NC
27	Test	Group R	M	67	Upper gingiva	Grade 3	T2N1	3	0.6	0	−1.93	S	NC
28	Test	Group R	M	53	Soft palate	Grade 3	T4N1	4A	8.14	0	−1.50	R	NC
29	Test	Group R	M	59	Buccal mucosa	Grade 2	T4N2b	4A	31.6	1	−1.38	R	NC
30	Test	Group R	M	53	Mandibula	Grade 1	T4N2b	4A	33.4	1	−1.27	R	NC
31	Test	Group R	M	68	Mandibula	Grade 2	T4N2b	4A	56.1	2	−1.20	S	NC
32	Test	Group R	F	54	Tongue	Grade 2	T2N2b	4A	37	1	−1.06	S	NC
33	Test	Group R	F	52	Lower gingiva	Grade 2	T4N2c	4A	60.8	2	−0.93	S	NC
34	Test	Group R	M	61	Retromolar trigone	Grade 1	T4N1	4A	40.7	1	−0.85	S	NC
35	Test	Group R	M	41	Tongue	Grade 3	T2N2c	4A	20.2	0	−0.79	R	NC
36	Test	Group R	M	66	Mandibula	Grade 1	T4N2b	4A	43	1	−0.72	S	NC
37	Test	Group R	M	74	Lower gingiva	Grade 2	T4N2b	4A	68	2	−0.51	S	NC
38	Test	Group R	M	69	Tongue	Grade 2	T3N2b	4A	25.8	0	−0.47	PF	NC
39	Test	Group R	M	57	Mandibula	Grade 1	T4N0	4A	31.8	0	−0.12	R	NC
40	Test	Group R	M	64	Tongue	Grade 2	T3N2c	4A	75	2	−0.10	PF	NC
41	Test	Group R	M	62	Maxila	Grade 2	T4N1	4A	33	0	−0.05	S/R	NC
42	Test	Group R	M	57	Tongue	Grade 2	T2N0	2	33.1	0	−0.04	S/R	NC
43	Test	Group R	M	64	Tongue	Grade 2	T3N2b	4A	72.4	1	0.99	S	NC
44	Test	Group S	M	57	Maxillary sinus	Grade 2	T3N0	3	0.7	2	−4.42	P/R	PR
45	Test	Group S	M	52	Lower gingiva	Grade 2	T2N2b	4A	1.35	2	−4.38	S/R	PR
46	Test	Group S	M	55	Tongue	Grade 3	T3N2b	4A	6.63	1	−2.83	PF	PR
47	Test	Group S	M	74	Upper gingiva	Grade 3	T4N2b	4A	9.94	1	−2.64	S/R	PR
48	Test	Group S	M	62	Tongue	Grade 3	T3N0	3	10.9	1	−2.58	S/R	PR
49	Test	Group S	M	71	Buccal mucosa	Grade 3	T3N2b	4B	39.9	2	−2.14	PF	PR
50	Test	Group S	F	76	Upper gingiva	Grade 2	T4N0	4A	0	0	−1.97	S/R	PR
51	Test	Group S	F	52	Tongue	Grade 3	T3N0	3	2.1	0	−1.85	S/R	PR
52	Test	Group S	M	50	Tongue	Grade 1	T2N0	2	6.2	0	−1.61	S/R	PR
53	Test	Group S	M	49	Tongue	Grade 2	T3N1	3	27.9	1	−1.60	PF	PR
54	Test	Group S	M	48	Lower gingiva	Grade 1	T4N0	4A	31.1	1	−1.41	S/R	PR
55	Test	Group S	M	62	Lower gingiva	Grade 2	T3N2b	4A	33.1	1	−1.29	S/R	PR
56	Test	Group S	M	58	Floor of mouth	Grade 2	T2N2c	4A	11.9	0	−1.28	R	PR
57	Test	Group S	M	61	Upper gingiva	Grade 3	T4N0	4A	12.2	0	−1.26	P/R	PR
58	Test	Group S	M	59	Floor of mouth	Grade 2	T4N2c	4A	33.7	1	−1.26	PF	PR
59	Test	Group S	F	67	Tongue	Grade 2	T2N2c	4A	34.8	1	−1.19	PF	PR
60	Test	Group S	F	64	Upper gingiva	Grade 2	T3N0	3	37.1	1	−1.06	S/R	PR
61	Test	Group S	M	58	Lower gingiva	Grade 2	T3N1	3	59	2	−1.03	S/R	PR
62	Test	Group S	M	54	Lower gingiva	Grade 2	T2N2b	4A	16.7	0	−1.00	S/R	PR
63	Test	Group S	M	69	Tongue	Grade 2	T3N0	3	20.8	0	−0.76	S/R	PR
64	Test	Group S	M	65	Tongue	Grade 1	T3N0	3	26.5	0	−0.43	PF	PR
65	Test	Group S	F	40	Tongue	Grade 2	T2N2b	4A	27.9	0	−0.35	S/R	PR
66	Test	Group S	M	54	Lower gingiva	Grade 2	T4N0	4A	28.3	0	−0.32	R	PR
67	Test	Group S	M	55	Tongue	Grade 1	T3N1	3	30.9	0	−0.18	S/R	PR
68	Test	Group S	M	59	Floor of mouth	Grade 2	T2N0	2	52.3	1	−0.17	S/R	PR
69	Test	Group S	M	67	Floor of mouth	Grade 2	T4N1	4A	31.1	0	−0.16	S/R	PR
70	Test	Group S	F	64	Lower gingiva	Grade 1	T4N1	4A	35.6	0	0.10	S/R	PR
71	Test	Group S	M	65	Tongue	Grade 1	T3N1	3	80	2	0.19	PF	PR
72	Test	Group S	M	66	Floor of mouth	Grade 2	T3N0	3	60.7	1	0.31	PF	PR
73	Test	Group S	M	65	Retromolar trigone	Grade 2	T2N0	2	41.4	0	0.44	R	PR
74	Test	Group S	M	75	Upper gingiva	Grade 3	T4N2c	4A	41.4	0	0.44	S/R	PR
75	Test	Group S	F	62	Maxila	Grade 1	T4N0	4A	42.6	0	0.51	PF/R	PR
76	Test	Group S	F	52	Tongue	Grade 2	T2N0	2	43.1	0	0.53	S/R	PR
77	Test	Group S	M	59	Tongue	Grade 1	T3N2c	4A	47	0	0.76	R	PR
78	Test	Group S	M	48	Tongue	Grade 2	T3N2b	4A	51.5	0	1.03	PF	PR
79	Test	Group S	M	62	Buccal mucosa	Grade 2	T2N0	2	52.8	0	1.10	S/R	PR
80	Test	Group S	F	46	Tongue	Grade 2	T3N0	3	54.3	0	1.18	S/R	PR
81	Test	Group S	M	45	Lower gingiva	Grade 1	T4N1	4A	54.4	0	1.19	S/R	PR
82	Test	Group S	M	45	Floor of mouth	Grade 1	T2N0	2	55.2	0	1.24	S/R	CR
83	Test	Group S	M	56	Tongue	Grade 3	T3N0	3	55.9	0	1.28	S/R	PR
84	Test	Group S	F	62	Lower Gingiva	Grade 2	T3N0	3	60.2	0	1.53	R	PR
85	Test	Group S	M	58	Soft palate	Grade 2	T3N0	3	69.9	0	2.09	S/R	PR
86	Test	Group S	M	68	Lower gingiva	Grade 1	T4N0	4A	71.6	0	2.19	S/R	PR

Group R, resistant group; Group S, sensitive group; M, male; F, female; Pre C/R, preoperative chemotherapy and/or radiotherapy; S/R, S-1 + radiation; P/R, carboplatin or cisplatin + radiation; PF/R, cisplatin and 5-fluorouracil + radiation; PF, cisplatin and 5-fluorouracil; S, S-1; R, radiation; CR, complete remission; PR, partial response; SD, stable disease; PD, progressive disease.

As learning cases, 18 biopsy samples were prepared (Table 1), including nine samples from Group R patients and nine samples from Group S patients. As test cases, 68 samples were prepared (Table 1), including 25 samples from Group R patients and 43 samples from Group S patients.

### Preoperative chemotherapy and/or radiotherapy

Patients assigned to the S/R group received a daily fractional dose of radiotherapy (2 Gy; 5 days/week) for a total dose of 34–50 Gy using a 4MV LINAC (Varian, CA). Radiation was delivered to the primary tumor site and the cervical nodes for patients with nodal involvement. Concomitant chemotherapy with S-1 (Taiho Pharmaceutical Co., Tokyo, Japan) was orally administered twice a day after a meal for five consecutive days per week. The individual doses were calculated on the basis of body surface area (total 1000–3000 mg).

Patients assigned to the P/R group received a fractional daily dose of radiotherapy for a total dose of 50 Gy. Concomitant chemotherapy with carboplatin (CBDCA) or cisplatin (CDDP) was administered once a day using the selective intra-arterial infusion method via the superficial temporal artery 9. The daily dose of CBDCA ranged from 10 to 30 mg, with a total dose of 495–725 mg. The daily dose of CDDP was 8 mg, with a total dose of 280 mg.

Patients in the pretreatment PF/R group received a daily fractional dose of radiotherapy for a total dose of 50 Gy. Concomitant chemotherapy with intravenous CDDP (80 mg/m^2^), followed by 5-fluorouracil (5-Fu) (800 mg/m^2^ per day) as a continuous 24-h infusion for five consecutive days, was administered.

Patients in the PF group received intravenous CDDP (60, 70, or 80 mg/m^2^), followed by 5-Fu (600, 700, or 800 mg/m^2^ per day) as a continuous 24-h infusion for five consecutive days. These patients received one or two cycles of treatment.

Patients in the pretreatment S group were administered oral S-1 twice a day after meals during the waiting period before surgery (total, 600–2100 mg).

Finally, patients in the pretreatment R group only received a daily fractional dose of radiotherapy (2.5 Gy for 4 days/week), with a total dose of 40 Gy.

### Peptide extraction

The proteins were extracted from the FFPE samples in the form of tryptic peptides using the Liquid Tissue MS Protein Partitioning Kit (Expression Pathology, Rockville, MD) according to the manufacturer's protocol. The extracted peptides were desalted through a C18 ZipTip (Millipore, Billerica, MA).

### Liquid chromatography–mass spectrometry

Eighteen samples (nine from Group R and nine from Group S) were blinded, randomized, and measured in duplicate with a linear gradient of 0–80% acetonitrile in 0.1% formic acid at a speed of 200 nL/min for 60 min using a nano-flow high-performance liquid chromatography (HPLC; NanoFrontier nLC; Hitachi High-technologies, Tokyo, Japan) that was connected to a triple time-of-flight mass spectrometer (5600 Triple TOF; AB Sciex, Framingham, MA). The system detected peptide peaks every 1 sec, with a mass-to-charge ratio (m/z) ranging from 400 to 1600. The MS peaks were detected, normalized, and quantified using in-house 2DICAL (two-dimensional image-converted analysis of liquid chromatography and mass spectrometry) software as described previously 10. A serial identification (ID) number was assigned to each MS peak from ID1 to ID70510.

### Protein identification by MS/MS

The MS/MS spectra were aligned with a tolerance of ±0.5 m/z and a retention time (RT) of ±0.4 min. Then, targeted MS/MS was performed. Peaks lists were generated by the MassNavigator software package (Version 1.2.12; Mitsui Knowledge Industry, Tokyo, Japan) and searched against the NCBInr database (NCBInr_20120423.fast) using the Mascot software package (Version 2.2.1; Matrix Sciences, London, UK). The initial peptide tolerances in MS and MS/MS modes were ±0.05 and ±0.1 Da, respectively. The peptides were digested with trypsin, and up to one missed cleavage was allowed. The score threshold for achieving *P* < 0.05 was set by the Mascot algorithm on the basis of database size. If a peptide matched multiple proteins, then the protein name with the highest Mascot score was selected.

### Immunohistochemistry for galectin-7

FFPE sections from all 86 cases were analyzed by quantitative immunohistochemistry (IHC). The 4-μm thick sections were incubated with a rabbit monoclonal anti-galectin-7 antibody (EPR4287; 1:2000; LifeSpan Biosciences, Inc., WA) for 60 min at room temperature. Then, they were incubated with biotinylated anti-primary antibodies (Histofine SAB PO kit; Nichirei, Tokyo, Japan). The signal was detected using streptavidin–peroxidase (Histofine SAB PO kit) and the diaminobenzidine tetrahydrochloride (DAB) substrate. Finally, the sections were counterstained with Mayer's hematoxylin and dehydrated.

### Analysis of galectin-7 immunostaining

The galectin-7-stained area (G7S) was quantified by dividing the staining intensity of that area over the staining intensity of the whole tissue section using ImageJ (freeware available at http://rsb.info.nih.gov/nih-image/). The immunostaining pattern of the galectin-7 nuclear staining area (G7N) was defined by dividing the staining intensity of the galectin-7 immunostained area within the nucleus over G7S. The median G7N of the 18 learning samples was 0.168. Therefore, G7NL was graded as follows: weak (G7NL = 0; <0.15 of G7N), positive (G7NL = 1; 0.15–0.40 of G7N), or strongly positive (G7NL = 2; >0.40 of G7N), corresponding to highest tertile of the 18 learning samples.

### Cell culture

The human oral SCC cell lines, HSC-3, HSC2, HOC313, HSC4, and Ho1-N-1, were established in the First and the Second Department of Oral and Maxillofacial Surgery, Faculty of Dentistry, Tokyo Medical and Dental University (Tokyo, Japan). SKN3 cells were purchased from the Japanese Collection of Research Bioresources (Osaka, Japan). HEK293 cells were purchased from DS Pharma Biomedical Co. Ltd. (EC85120602-FO; Osaka, Japan). These were maintained in Dulbecco's modified Eagle's medium (DMEM) containing a high concentration of glucose (4.5 mg/mL) supplemented with 10% fetal bovine serum (FBS) and cultured in a 5% CO_2_ environment at 37°C.

### Adenovirus vector

A cDNA for human *galectin-7* (Clone name: pFN21AE1213) was obtained from the Kazusa DNA Research Institute, Kisarazu, Japan (http://www.kazusa.or.jp). The adenoviral construct containing FLAG-tagged human galectin-7 (GAL7) was obtained using Adeno-X™ Adenoviral System 3 with tetracycline inducible expression system (Tet-On 3G Inducible) from Clontech (Mountain View, CA). FLAG (ATGGACTACAAGGACGACGATGACAAG) and human *galectin-7* sequences can be transferred as PCR products to the pAdenoX vector using the In-Fusion^**®**^ cloning method (Clontech) according to the protocol. FLAG-tagged human *galectin-7* gene plus 15 bp of homology to pAdenoX vector was amplified using CloneAmp HiFi Premix (Clontech) with the following primers: 5′-GTAACTATAACGGTCATGGACTACAAGGACGACGATGACAAGATGTCCAACGTCCCCCACAAGTCCT-3′ (Ad-FLAG-GAL7 forward), 5′-ATTACCTCTTTCTCCTCAGAAGATCCTCACGGAGTCCAGCT-3′ (GAL7 reverse). The Pac I-Digested adenoviral construct was transfected into HEK293 cells. The virus was amplified and harvested according to the Clonetech protocol. The viral titer was determined by the Tissue Culture Infectious Dose 50 (TCID50) method. The infection was with the multiplicity of infection (MOI) of 0–100 IFU/cell in complete growth medium with or without 1 *μ*g/mL doxycycline (Clontech).

### Western blot analysis

Cells were lysed in a cell lysis buffer (20 mmol/L Tris-HCl pH 7.5, 1% Triton X-102, 0.1% SDS, 150 mmol/L NaCl, 1 mmol/L EDTA, 50 *μ*g/mL Aprotinin and 80 *μ*g/mL PMSF). The cell lysate was subjected to SDS-PAGE (sodium dodecyl sulfate polyacrylamide gel electrophoresis), followed by electroblotting onto polyvinylidene fluoride (PVDF) membranes. After blocking with 5% skim milk in phosphate-buffered saline (PBS) for 1 h, membranes were probed with specific a mouse monoclonal anti-FLAG (M2; 1:1000; Sigma, St. Louis, MO), a rabbit monoclonal anti-galectin-7 (EPR4287; 1:1000; LifeSpan Biosciences, Inc.), a rabbit polyclonal anti-caspase-3 (#9662; 1:1000; Cell Signaling Technology, Inc., MA), and a rabbit monoclonal anti-beta-actin (#8457(D6A8); 1:1000; Cell Signaling Technology, Inc.) antibodies. Immunoreactive signals were visualized by SuperSignal^**®**^ west dura extended duration (Thermo Scientific, Waltham, MA), using Light-capture^**®**^ (ATTO, Bioscience and Biotechnology, Tokyo, Japan).

### In vitro cell viability assay

Cell viability was assessed by the colorimetric water-soluble tetrazolium salt (WST) assay (Cell counting kit-8; Dojindo Laboratories, Kumamoto, Japan) as described by the manufacturer. Briefly, cells (1 × 10^4^/well) were seeded into 96-well plates and cultured for 12 h. Then, cells were incubated with adenovirus infection for 12 h with or without 1 *μ*g/mL doxycycline, and further cultured with or without doxycycline in fresh medium with the absence or presence of anticancer drug, 5 *μ*g/mL cisplatin or 50 *μ*g/mL 5-fluorouracil, for an additional 24 h. At the end of the culture period, 10 *μ*L of a formazan-generating reagent, WST-8, was added to each well for 60 min at 37°C. The absorbance of each well at 450 nm was then measured using a microplate reader.

### Statistical Analysis

The MS data and cell viability were analyzed by receiver operating characteristic (ROC) curve analysis, including area under the curve (AUC), stepwise discriminant analysis, *T*-tests, Mann–Whitney *U*-tests, and two-sided log-rank tests with PASW Statistics, version 18 (SPSS Inc., Chicago, IL). *P *<* *0.05 was considered statistically significant.

## Results

### Identification of a predictive marker of chemotherapy and/or radiotherapy resistance in OSCC by LC-MS

**Figure 1:**
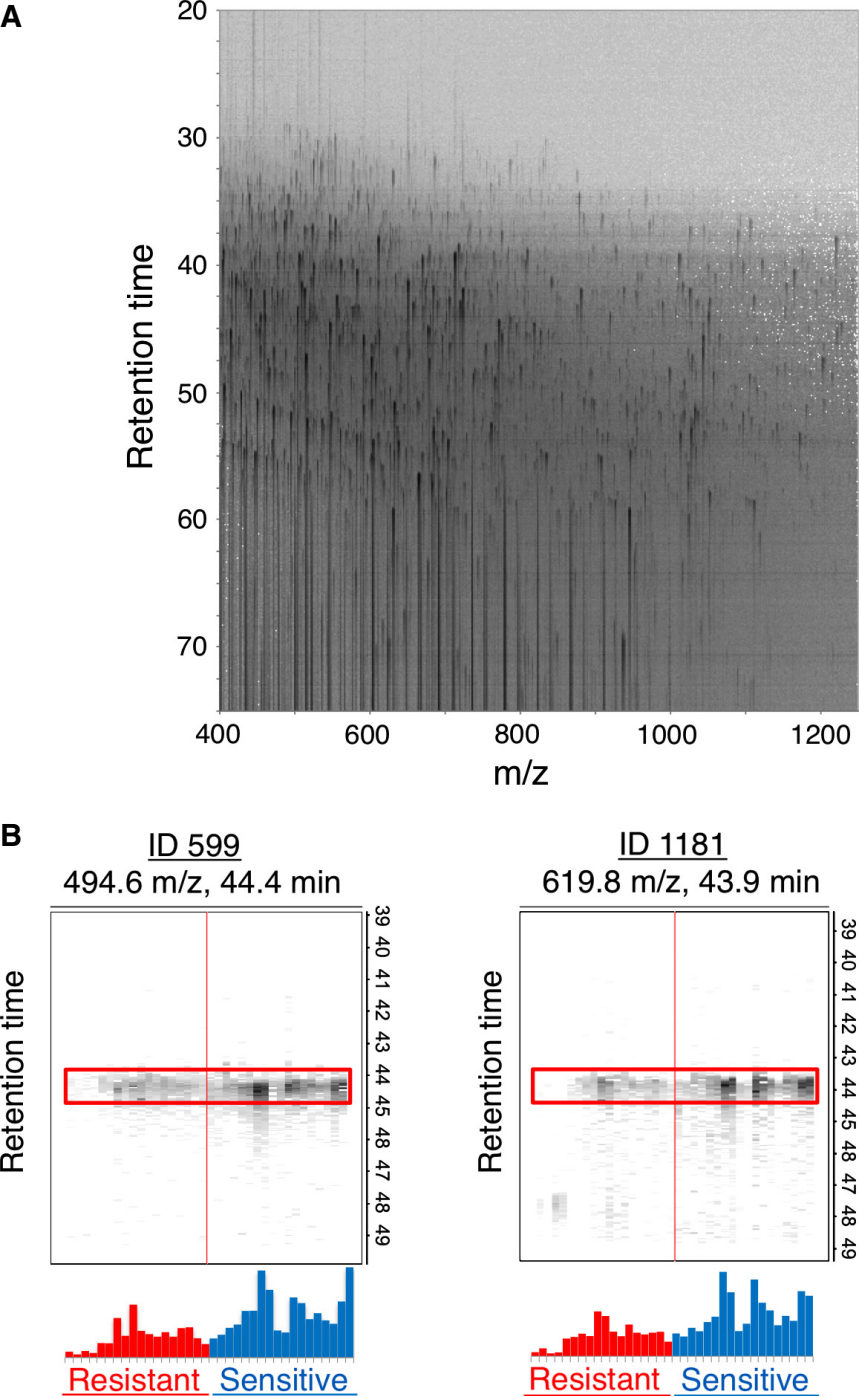
Identification of proteins differentially expressed in OSSC samples from patients sensitive (Group S) or resistant (Group R). The samples were processed by LC-MS and analyzed with 2DICAL. (A) Two-dimensional display of a representative sample. (B) Separation gel for the MS peaks ID599 and ID1181 from each group (*N* = 18). Group R (red) exhibits lower expression of the two peaks compared with Group S (blue).

To identify a predictive marker of chemotherapy and/or radiotherapy resistance in OSCC, 18 biopsy samples, including nine samples from Group R and nine samples from Group S, were analyzed by LC-MS (Fig. 1). A total of 70,510 peaks per sample were readily detected and quantified. Among them, 10,869 MS peaks differed in intensity between Group R and Group S (*P *<* *0.05; AUC >0.7). We further selected 105 peaks with Mascot scores >50 and excluded abundant proteins, including keratin, fibrinogen, collagen, and histone. Among them, we selected 20 peaks with Group R/Group S peak intensity ratios ≤0.5. We further limited our selection to the peaks with high predictive power on the basis of discriminant analysis (stepwise method), which identified two candidate peaks (ID1181 and ID2504). We chose the peak with the highest Mascot score: ID1181 (Table 2). MS/MS spectra of the ID1181 peaks matched the amino acid sequence of the galectin-7 protein.

**Table 2:** List of peptides that differed between the resistant group and sensitive group.

ID	M/Z	RT	Charge	Ratio (R/S)	UniProt accession number	Protein Description	Mascot score	Peptide sequence	Resistant (mean ± SD)	Sensitive (mean ± SD)
4722	725.7	55.3	3	0.47	P31947	14-3-3 protein sigma	62.41	TTFDEAMADLHTLSEDSYK	1070 ± 538	2263 ± 846
6326	776.4	55.3	2	0.48	P04083	Annexin A1	62.79	GTDVNVFNTILTTR	2782 ± 1387	5762 ± 3858
25210	574.0	38.9	3	0.25	P22528	Cornifin-B	51.67	QPCTPPPQLQQQQVK	71 ± 83	282 ± 293
1294	726.7	57.3	3	0.48	P15924	Desmoplakin	129.32	FLEFQYLTGGLVDPEVHGR	1805 ± 1148	3765 ± 2017
2504	627.8	50.5	2	0.39	P15924	Desmoplakin	60.97	AITGFDDPFSGK	958 ± 348	2460 ± 938
3011	843.5	50.8	2	0.45	P15924	Desmoplakin	116.28	ITNLTQQLEQASIVK	846 ± 351	1889 ± 751
3292	752.1	55.7	3	0.48	P15924	Desmoplakin	105.46	LLEAQIASGGVVDPVNSVFLPK	1109 ± 568	2304 ± 930
1196	677.4	52.3	2	0.47	P12724	Eosinophil cationic protein	63.88	YPVVPVHLDTTI	1297 ± 443	2786 ± 1933
599	494.6	44.4	3	0.45	P47929	Galectin-7	52.89	SSLPEGIRPGTVLR	2730 ± 1558	6018 ± 2658
1181	619.8	43.9	2	0.47	P47929	Galectin-7	87.57	LDTSEVVFNSK	1763 ± 993	3764 ± 1639
31232	593.6	48.5	2	0.42	P01857	Ig gamma-1 chain C region	57.90	GPSVFPLAPSSK	192 ± 225	453 ± 169
1316	618.9	51.4	2	0.46	P14923	Junction plakoglobin	87.37	VSVELTNSLFK	1815 ± 1296	3931 ± 1850
914	576.8	52.3	2	0.42	P05164	Myeloperoxidase	56.64	IANVFTNAFR	925 ± 727	2198 ± 1956
20233	576.8	52.7	2	0.48	P05164	Myeloperoxidase	56.77	IANVFTNAFR	659 ± 404	1365 ± 778
2998	688.9	46.5	2	0.47	Q13835	Plakophilin-1	66.93	VMGNQVFPEVTR	1035 ± 781	2190 ± 1187
10288	594.3	37.7	2	0.42	Q13835	Plakophilin-1	85.89	LLQSGNSDVVR	413 ± 235	987 ± 597
26579	450.7	36.8	2	0.49	Q13835	Plakophilin-1	50.69	LDAEVPTR	261 ± 181	538 ± 346
1257	582.0	46.1	3	0.48	P06702	Protein S100-A9	65.32	VIEHIMEDLDTNADK	1365 ± 945	2817 ± 1354
2735	807.9	66.0	2	0.48	P06702	Protein S100-A9	111.26	QLSFEEFIMLMAR	829 ± 807	1743 ± 1423
9832	744.3	42.5	2	0.49	P68363	Tubulin alpha-1B chain	87.43	LISQIVSSITASLR	426 ± 143	870 ± 572

RT, retention time; Ratio (R/S), a ratio of average peak intensity in the resistant group to that in the sensitive group.

### Expression of galectin-7 in OSCC by IHC

**Figure 2:**
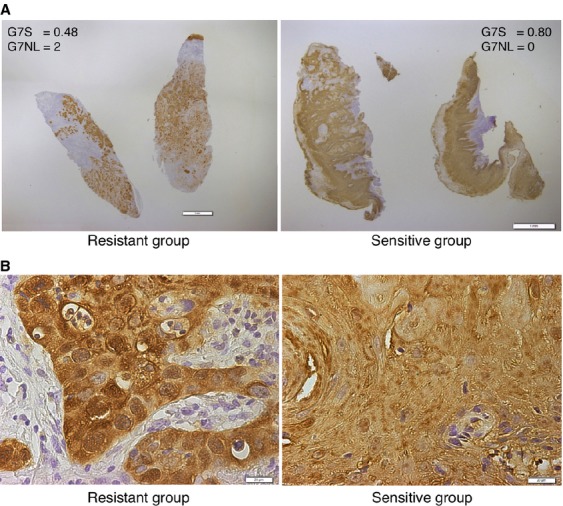
Immunohistochemistry of galectin-7. (A) Low magnification (bar: 1 mm) showing the overall staining intensity and distribution. Left panel: galectin-7 staining area (G7S) = 0.48, galectin-7 nuclear staining level (G7NL) = 2. Right panel: G7S = 0.80, G7NL = 0. (B) High magnification (bar: 20 *μ*m) showing galectin-7 was expressed in both the cytosolic and nuclear compartments; we observed strong nuclear staining in Group R and mostly cytosolic staining in Group S.

**Figure 3:**
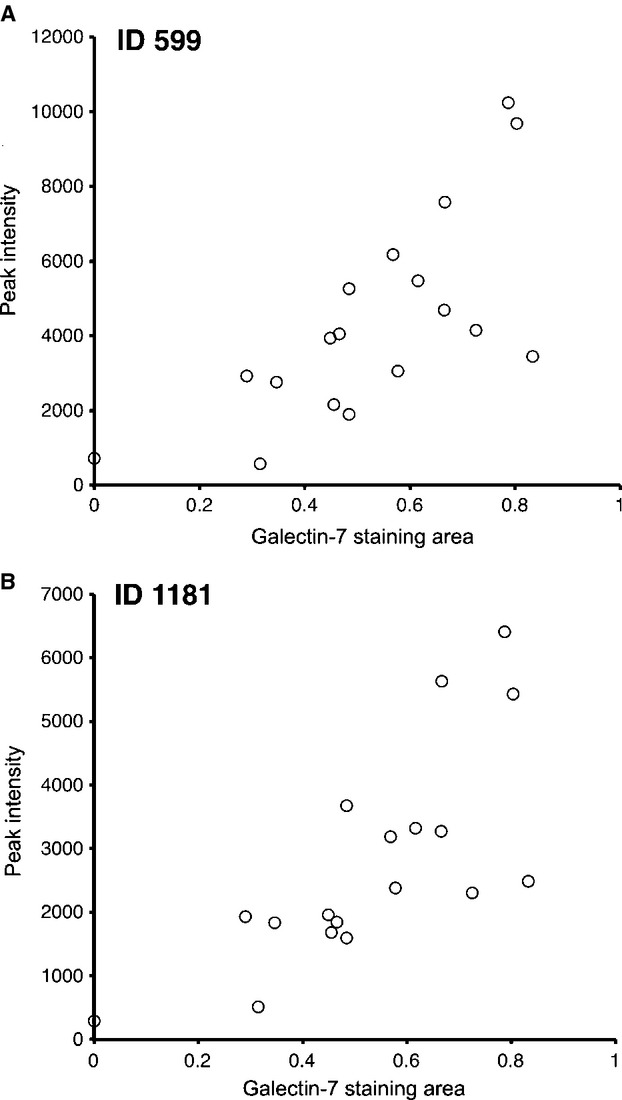
Regression analysis between the galectin-7 staining area (G7S) and the intensity of the MS peaks. Significant positive correlations were detected for both peaks, namely (A) ID599 (*r *=* *0.71; *P *=* *0.001) and (B) ID1181 (*r *=* *0.73; *P *=* *0.001).

**Figure 4:**
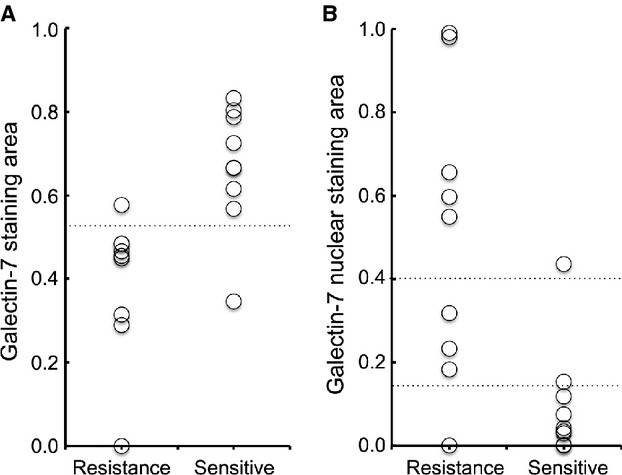
Scatter plot analysis for galectin-7 immunostaining. The two groups were compared for quantitative values of (A) galectin-7 staining area (G7S) and (B) galectin-7 nuclear area (G7N). Median G7S was lower for Group R than for Group S, but median G7N was 10-fold higher for Group R than Group S. Classification values for the galectin-7 nuclear staining area are shown by the horizontal lines as values of 0.15 and 0.40.

The protein expression of galectin-7 in OSCC tissue was confirmed by IHC analysis of the FFPE samples used for LC-MS analysis (Fig. 2A). Pearson's correlation analysis was conducted between G7S and each of the peaks listed in Table 2. A significant positive correlation was detected between G7S and the intensity of the two galectin-7 peaks, namely ID599 (*r *=* *0.71; *P *=* *0.001; Fig. 3A) and ID1181 (*r *=* *0.73; *P *=* *0.001; Fig. 3B). Scatter plot analysis was conducted for the G7S values of Group R and Group S (Fig. 4A). The median G7S was significantly lower for Group R (0.455) than for Group S (0.666; Mann–Whitney *U*-test; *P *=* *0.003), suggesting that oral SSC patients with low galectin-7 expression are more likely to exhibit chemotherapy and/or radiotherapy resistance. The sensitivity of this prediction was 88.9% and specificity was 88.9% (cutoff point: 0.526).

A careful observation of the IHC findings revealed that galectin-7 was expressed in both the cytosolic and nuclear compartments; we observed strong nuclear staining in Group R and mostly cytosolic staining in Group S (Fig. 2). Median G7N was 10-fold higher for Group R than for Group S, with 0.549 and 0.042, respectively (Mann–Whitney *U*-test; *P *=* *0.003; Fig. 4B). These data show that chemotherapy and/or radiotherapy resistance is associated with a nuclear concentration of galectin-7. Therefore, we conducted a discriminant analysis using G7S and G7NL for the 18 learning samples analyzed by LC-MS and IHC and obtained the following predictive formula for chemotherapy and/or radiotherapy resistance:


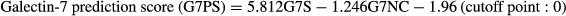


**Figure 5:**
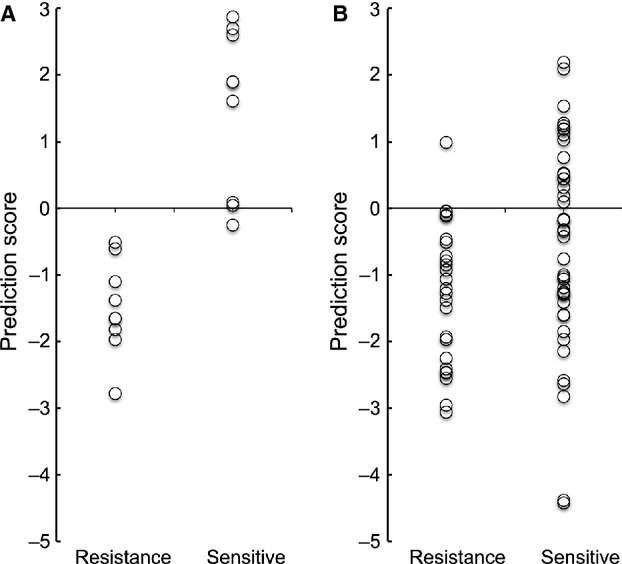
Scatter plot analysis for the galectin-7 prediction score (G7PS). (A) In the learning cases. (B) In the test cases.

Based on this formula, the sensitivity of prediction was 100% and specificity was 88.9%, indicating that sensitivity was increased in the 18 learning cases (Fig. 5A). When this formula was used to analyze the remaining 68 test cases, the sensitivity of prediction was 96.0% and specificity was 39.5% (Fig. 5B).

### Galectin-7 prediction score correlates with poor prognosis in patients with OSCC

**Figure 6:**
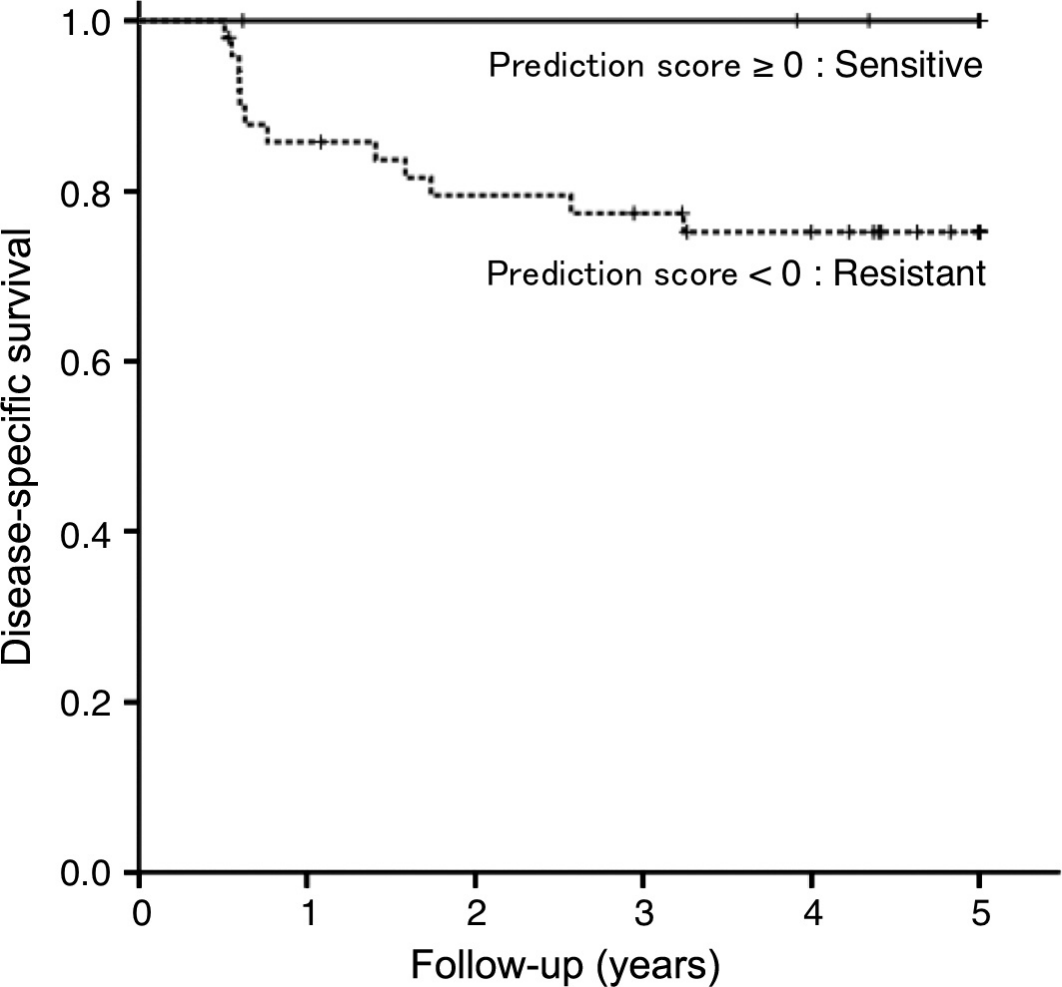
Kaplan–Meier survival analysis based on G7PS. There was a significant correlation between resistant prediction and survival parameter (log-rank test; *P *=* *0.027).

Five-year cumulative survival rates in Group S and Group R were estimated by Kaplan–Meier analysis using galectin-7 as a predictor of chemotherapy and/or radiotherapy resistance. The cumulative 5-year disease-specific survival rate was 75.2% in patients with resistant prediction using galectin-7 prediction score (G7PS) (<0) and 100% in patients with sensitive prediction (G7PS ≥0; Fig. 6). There was a significant positive correlation between resistant prediction using G7PS and survival parameter (log-rank test; *P *=* *0.027; Fig. 6).

### Galectin-7 decreases cell viability

**Figure 7:**
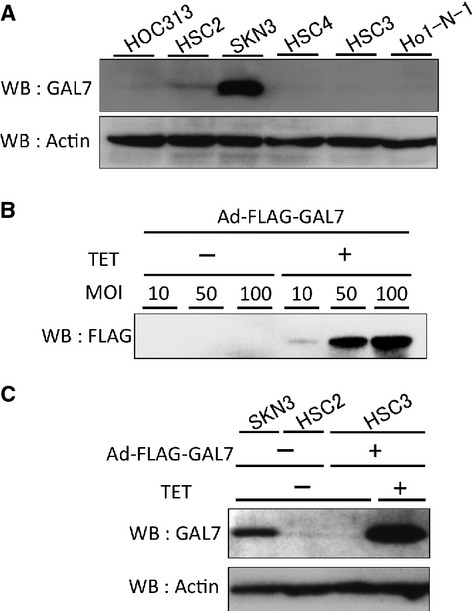
Adenovirus-mediated expression of galectin-7 in oral SCC cell lines. (A) Endogenous expression of galectin-7 in oral SCC cell lines. (B) Adenovirus-mediated expression of galectin-7 in HSC3 cells. HSC3 cells were infected with the indicated MOI of a recombinant adenovirus encoding FLAG-tagged galectin-7 (Ad-FLAG-GAL7) with or without 1 *μ*g/mL doxycycline. (C) FLAG-tagged galectin-7 in HSC3 cells strongly expressed rather than endogenous expressions of galectin-7 in SKN3 or HSC2 cells. HSC3 cells were infected with MOI 50 of Ad-FLAG-GAL7. Then, SKN3, HSC2 and HSC3 cells were cultured with or without doxycycline in fresh medium for 24 h. The lysates were analyzed by Western blot analysis.

**Figure 8:**
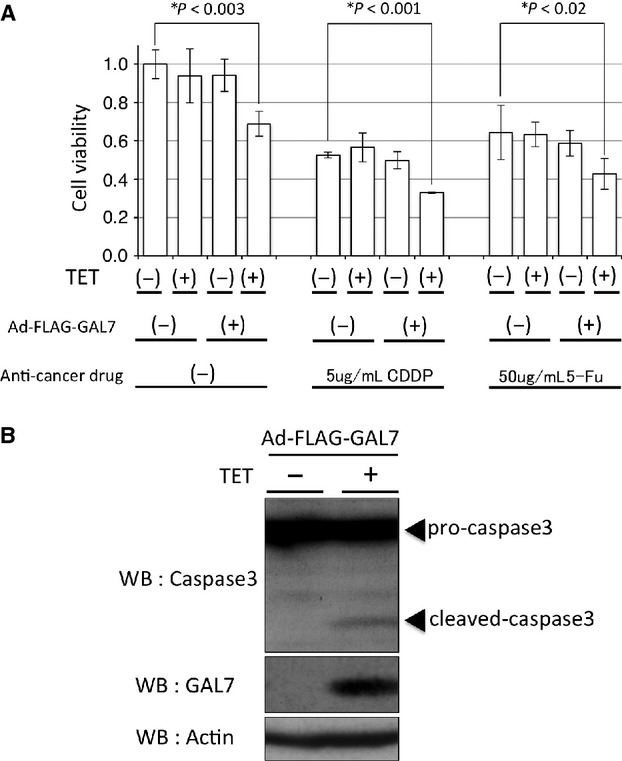
Overexpression of galectin-7 decreased cell viability. (A) Effect of galectin-7 in HSC3 cells. HSC3 cells were infected with Ad-FLAG-GAL7 at MOI 50. Then, cells were cultured with or without doxycycline in a fresh medium with the absence or presence of anticancer drug, 5 *μ*g/mL cisplatin or 50 *μ*g/mL 5-fluorouracil. Cell viability was assayed as described in. Data represent the means ± SD of four independent experiments. *P <* *0.05, compared with uninfected cells in the absence of anticancer drugs. 5-Fu, 5-fluorouracil; CDDP, cisplatin; TET, doxycycline. (B) Cleavage of caspase-3 induced by the overexpression of galectin-7. HSC3 cells were infected with Ad-FLAG-GAL7 at MOI 50. Then, cells were cultured with or without doxycycline in a serum-free medium for 36 h. The lysates were analyzed by Western blot analysis. Beta-actin was used as a loading control.

To investigate the roles of galectin-7 in OSCC cells, the expression status of galectin-7 in six human OSCC cell lines was detected by Western blot analysis. A low endogenous expression of galectin-7 was detected in all OSCC cell lines except SKN3 (Fig. 7A). Next, we examined the effect of overexpressed galectin-7 in OSCC cells. HSC3 cells were infected with recombinant adenovirus encoding FLAG-tagged galectin-7 (Ad-FLAG-GAL7). The expression of galectin-7 was detected in a MOI-dependent manner with 1 *μ*g/mL doxycycline by Western blot analysis (Fig. 7B), and we confirmed that FLAG-tagged galectin-7 was strongly expressed in HSC3 cells than endogenous expressions of galectin-7 in SKN3 or HSC2 cells (Fig. 7C). To examine the infection efficiency and intracellular distribution of Ad-FLAG-GAL7, we performed immunofluorescence labeling for overexpressed galectin-7. The infection efficiency of Ad-FLAG-GAL7 in HSC3 cells at MOI 50 was ∼80% (Fig. S1A). The intracellular distribution of Ad-FLAG-GAL7 was similar to the IHC staining pattern of galectin-7 (Fig. 8B). Moreover, by Western blot analysis, we confirmed that analysis of supernatants from HSC3 cells infected with Ad-FLAG-GAL7 or other OSCC cell lines have failed to provide evidence for a secreted form of galectin-7 (data not shown). To examine the effect of galectin-7 on cell viability, HSC3 cells infected with Ad-FLAG-GAL7 (MOI of 50) were cultured for 24 h with or without doxycycline. The overexpression of galectin-7 significantly decreased cell viability in normal culture conditions (Fig. 8A). Furthermore, similar results were observed when we treated the cells with 5 *μ*g/mL cisplatin or 50 *μ*g/mL 5-fluorouracil (Fig. 8A). These results indicate that galectin-7 may be involved in tumor cell proliferation/viability rather than chemosensitivity. We also investigated the role of galectin-7 using antisense galectin-7 oligonucleotides in OSCC cell lines. Unfortunately, the results showed no effects of galectin-7 knockdown on cell viability (Fig. S2). To determine whether the decreased cell viability was because of apoptosis, Ad-FLAG-GAL7-infected HSC3 cells were cultured with or without doxycycline. We observed weak activation and cleavage of caspase-3 induced by the overexpression of galectin-7 was observed (Fig. 8B) indicating that the decreased cell viability by overexpression of galectin-7 may be because of growth arrest rather than apoptosis.

## Discussion

The identification of tumor response predictors applying to a variety of radiotherapy and chemotherapy regimens will allow oncologists to customize strategies aimed at minimizing exposure to high-dose adjuvant therapy. To identify candidate biomarkers from the proteomics data, we used the 2DICAL analysis platform that performs a quantitative comparison of unlabeled shotgun proteomics data generated by LC-MS 11. This approach was successfully used to identify blood biomarkers in pancreatic and colorectal cancer patients 12–17. The use of proteins extracted from FFPE tissues in the form of tryptic peptides is compatible with a variety of MS-based proteomics 8. However, we present the first report, as per our knowledge, on tumor response predictors in patients with OSCC.

Galectins constitute a family of evolutionary-conserved carbohydrate-binding proteins. They are widely distributed in all organisms and are implicated in many essential functions such as development, differentiation, cell–cell adhesion, cell–matrix interaction, growth regulation, and apoptosis 18. The various roles of galectins in cancer invasion and metastasis have been well documented 19,20. However, very little is known about how galectin-7 expression affects cancer progression 18. The expression level of galectin-7 varies widely among tumor types, from completely downregulated to highly upregulated 21–24. De novo galectin-7 expression by p53 is associated with apoptosis, which inhibits tumor growth 25. Alternatively, galectin-7 mediates the nuclear export of Smad3 through complex formation, which is essential for the tumor-suppressive effects of hepatocyte growth factor on the transforming growth factor-beta signaling pathway 26. Paradoxically, galectin-7 may also induce the expression of genes known to promote cancer progression, including matrix metalloproteinase-9 (MMP-9) 27,28. In this case, galectin-7 expression could be induced by nuclear factor-kappa B, a transcription factor and a positive regulator of MMP-9 known to be expressed in highly aggressive tumor cells 18. This study provides in vitro evidence suggesting not a promoting effect of galectin-7 on the chemosensitivity but a potential of galectin-7 on decreasing cell proliferation/viability, which may be an interesting topic for future studies on OSCC therapy. We also investigated the role of galectin-7 using antisense galectin-7 oligonucleotides, however, the results showed no effects of galectin-7 knockdown on cell viability. A plausible reason for this is that strongly overexpressed galectin-7 affected cell viability, whereas physiologically expressed galectin-7 did not. Therefore, the knockdown of physiologically expressed galectin-7 did not elicit a noticeable effect. Moreover, because the function of galectin-7 in the nucleus remains unclear, further studies are needed to clarify this issue.

This study suggests that low galectin-7 protein expression translates into low cumulative survival rate in patients with OSCC via tumor resistance to preoperative chemotherapy and/or radiotherapy. These data are not consistent with the findings of Saussez et al. 29, who reported that galectin-7 protein expression increases during tumor progression in hypopharyngeal and laryngeal SCC. However, this research team also reported that the percentage of cells immunopositive for galectin-7 decreased significantly with the loss of histological differentiation in hypopharyngeal SCC 30. Furthermore, tumor progression was associated with a translocation of galectin-7 from the nucleus to the cytoplasm 29. These data suggest that the sequestration of galectin-7 into the nucleus would prevent this protein from interfering with tumor progression or regression, leading to treatment resistance. Accordingly, a formula combining the expression level and nucleic/cytoplasmic expression ratio of galectin-7 would constitute a rational predictor of chemotherapy and/or radiotherapy resistance in patient with advanced OSCC.

Our findings suggest that the specificity of the prediction of chemotherapy and/or radiotherapy resistance using G7PS was 39.5%; therefore, there could be many false-positive results. However, one case with a response evaluation of PR who was predicted as resistant (G7PS = −0.25) died of uncontrolled neck node metastases despite surgery followed by additional radiotherapy. Therefore, patients with resistance prediction using G7PS (<0) should be considered for other treatments or drugs that were not used in this study (e.g., docetaxel or a molecular target drug). Moreover, a synergistic effect of galectin-7 and S100-A9 on cervical squamous carcinoma patients was recently reported 31. We also identified S100-A9 as one of other potential predictive markers; therefore, identification of other useful combination markers that were not analyzed in this study may be important.

In conclusion, we identified galectin-7 as a predictor of tumor resistance and developed a predictive formula for patient survival. Accordingly, measurement of galectin-7 protein expression in fresh tissue biopsy samples at the time of diagnosis can provide invaluable information for the design of customized preoperative treatment regimens with advanced OSCC. Future challenges include the identification of other useful combination markers that were not analyzed in this study in order to improve diagnostic accuracy.

## Supporting Information

Figure S1 Infection efficiency and intracellular distribution of Ad-FLAG-GAL7. HSC3 cells were plated onto cell culture slide, infected with Ad-FLAG-GAL7, and cultured overnight with or without 1 μg/mL doxycycline. After a brief wash in PBS, the cells were fixed in 4% paraformaldehyde in PBS for 20 min at RT. After several washes in PBS, the cells were permeabilized and blocked in PBS containing 0.1% Triton X-102 and 3% bovine serum albumin (BSA) for 30 min. They were then incubated with a rabbit monoclonal anti-galectin-7 antibody (EPR4287; 1:200; LifeSpan Biosciences, Inc.) in PBS for 1 h at RT, followed by three washes with PBS. Samples were incubated for 45 min with a goat anti-rabbit IgGTR antibody (sc-2780; 1:100; Santa Cruz Biotechnology, Inc., CA), washed three times with PBS, and the slides were mounted in 90% glycerol. Samples were then analyzed and fluorescence images were recorded using a Zeiss Axiovert 135 Fluorescence Microscope (Carl Zeiss, Oberkochen, Germany). (A) 100×magnification, bar: 100 μm. Infection efficiency was approximately 80% in HSC3 cells at MOI 50. (B) 4009 magnification, bar: 20 μm. Intracellular distribution of Ad-FLAG-GAL7 is similar to IHC staining pattern of galectin-7.

Figure S2 In vitro cell viability in galectin-7 knockdown HSC2 cells. Galectin-7 (sc-44534-V) shRNA lentiviral particles and scramble control shRNA lentiviral particles-A (sc-108080) were purchased from Santa Cruz Biotechnology. Lentiviral transduction was performed in HSC2 cells. Pools of stable transductants were generated via selection with puromycin (10 μg/mL) by the manufacturer's protocol. HSC2 cells stably transduced with galectin-7-shRNA (AS) or scramble control shRNA (Cont.) were cultured in a growing medium for 60 h. (A) In vitro cell viability was determined with WST assays. No effects of galectin-7 knockdown on cell viability were observed. (B) The lysate was analyzed by Western blot analysis. Beta-actin was used as a loading control.
