# Supplementary figures and images for: Galectin-7 as a potential predictive marker of chemo-and/or radio-therapy resistance in oral squamous cell carcinoma
Source: Cancer Med. 2014 Feb 7;3(2):349–61. doi: 10.1002/cam4.195 (PMC3987084; doi:10.1002/cam4.195)

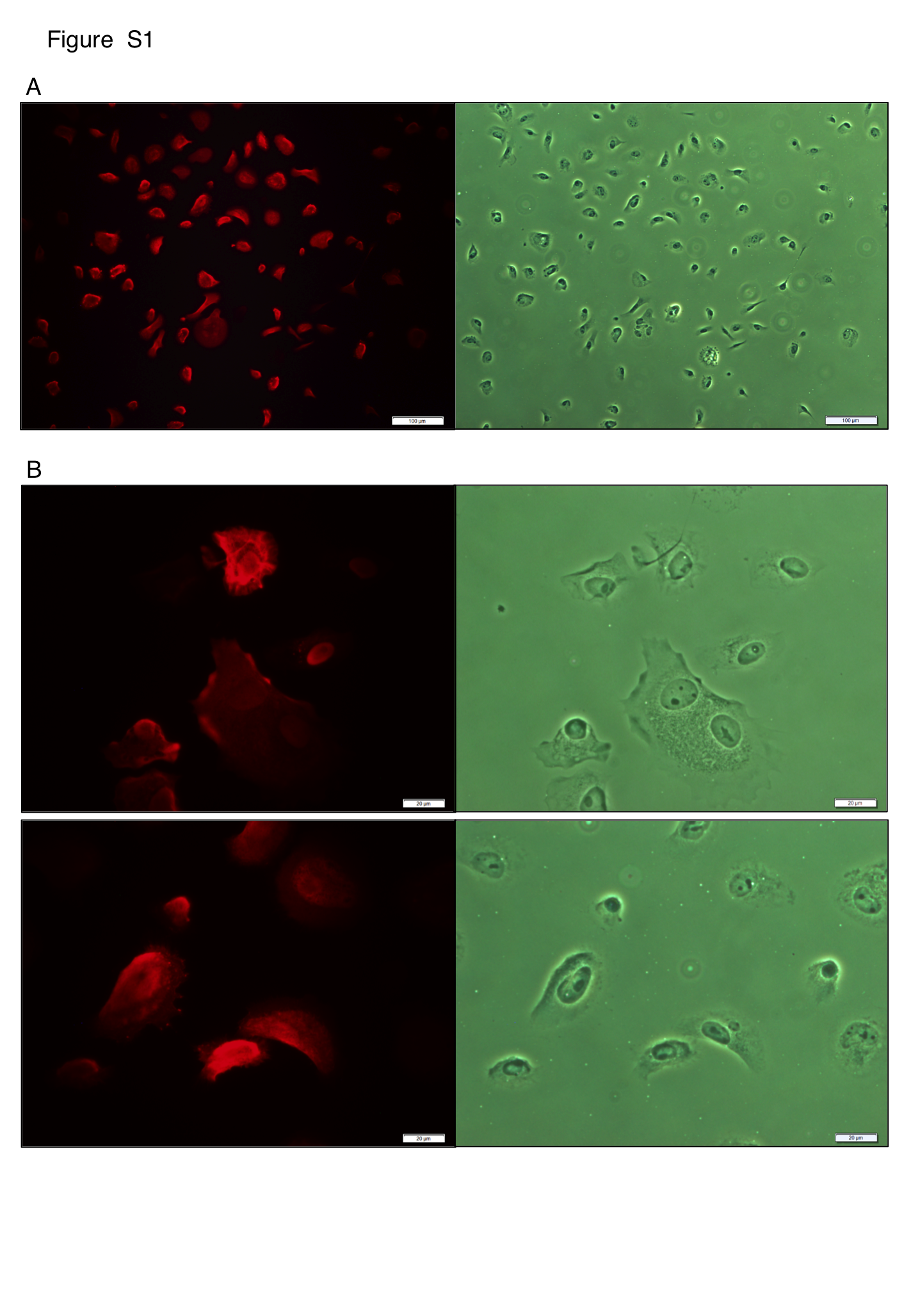

Supplement: Figure S1 — Infection efficiency and intracellular distribution of Ad-FLAG-GAL7. HSC3 cells were plated onto cell culture slide, infected with Ad-FLAG-GAL7, and cultured overnight with or without 1 μg/mL doxycycline. After a brief wash in PBS, the cells were fixed in 4% paraformaldehyde in PBS for 20 min at RT. After several washes in PBS, the cells were permeabilized and blocked in PBS containing 0.1% Triton X-102 and 3% bovine serum albumin (BSA) for 30 min. They were then incubated with a rabbit monoclonal anti-galectin-7 antibody (EPR4287; 1:200; LifeSpan Biosciences, Inc.) in PBS for 1 h at RT, followed by three washes with PBS. Samples were incubated for 45 min with a goat anti-rabbit IgGTR antibody (sc-2780; 1:100; Santa Cruz Biotechnology, Inc., CA), washed three times with PBS, and the slides were mounted in 90% glycerol. Samples were then analyzed and fluorescence images were recorded using a Zeiss Axiovert 135 Fluorescence Microscope (Carl Zeiss, Oberkochen, Germany). (A) 100×magnification, bar: 100 μm. Infection efficiency was approximately 80% in HSC3 cells at MOI 50. (B) 4009 magnification, bar: 20 μm. Intracellular distribution of Ad-FLAG-GAL7 is similar to IHC staining pattern of galectin-7. [file cam40003-0349-sd1.tiff]

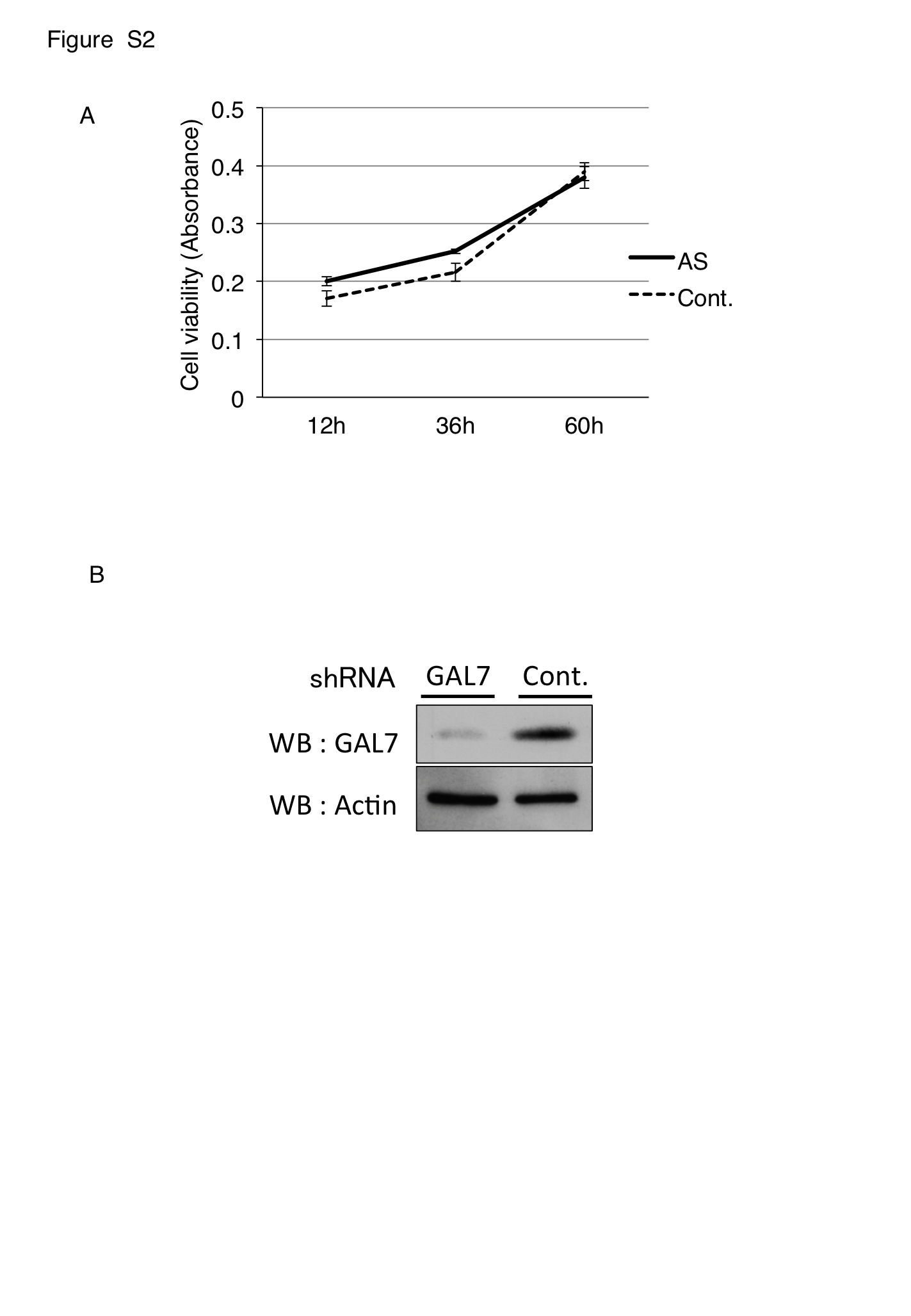

Supplement: Figure S2 — In vitro cell viability in galectin-7 knockdown HSC2 cells. Galectin-7 (sc-44534-V) shRNA lentiviral particles and scramble control shRNA lentiviral particles-A (sc-108080) were purchased from Santa Cruz Biotechnology. Lentiviral transduction was performed in HSC2 cells. Pools of stable transductants were generated via selection with puromycin (10 μg/mL) by the manufacturer's protocol. HSC2 cells stably transduced with galectin-7-shRNA (AS) or scramble control shRNA (Cont.) were cultured in a growing medium for 60 h. (A) In vitro cell viability was determined with WST assays. No effects of galectin-7 knockdown on cell viability were observed. (B) The lysate was analyzed by Western blot analysis. Beta-actin was used as a loading control. [file cam40003-0349-sd2.tiff]
